# Biological Therapy in Primary Sjögren's Syndrome: Effect on Salivary Gland Function and Inflammation

**DOI:** 10.3389/fmed.2021.707104

**Published:** 2021-07-15

**Authors:** Farzana Chowdhury, Anwar Tappuni, Michele Bombardieri

**Affiliations:** ^1^Centre for Experimental Medicine and Rheumatology, William Harvey Research Institute, London, United Kingdom; ^2^Institute of Dentistry, Barts and the London School of Medicine and Dentistry, London, United Kingdom

**Keywords:** B cell depletion, B/T cell co-stimulation, secretory function, salivary gland histopathology, salivary gland ultrasound

## Abstract

Primary Sjögren's syndrome (pSS) is a chronic, systemic autoimmune disease. It is the second most common rheumatic autoimmune disorder, affecting 0.7% of European Americans and up to 1% of people globally. pSS is characterized by the impaired secretory function of exocrine glands, including salivary and lachrymal glands. A lymphocytic infiltration of these organs leads to the common and debilitating symptoms of oral and ocular dryness, majorly affecting the quality of life of these patients. Currently, no disease-modifying drug has been approved for the treatment of pSS, with therapies largely aimed at relieving symptoms of dry mouth and dry eyes. In particular, management of oral dryness still represents a major unmet clinical need in pSS and a significant burden for patients with this condition. Recently, several randomized clinical trials in pSS with biological therapies targeting specific mechanistic pathways implicated in the disease pathogenesis, including B-cell hyperactivity, T-cell co-stimulation and the aberrant role of cytokines, have been completed with mixed results. In this review, we summarize evidence from recent clinical trials investigating biological therapy in pSS, specifically highlighting efficacy, or lack thereof, in modulating local inflammation and improving salivary gland function.

## Introduction

Primary Sjögren's syndrome (pSS) is described as a systemic autoimmune disease targeting the exocrine glands, mainly the salivary and lachrymal glands. A focal lymphocytic infiltration around the intercalated and striated ducts of these organs results in a progressive loss of glandular secretory function, resulting in debilitating sicca symptoms of xerostomia (dry mouth) and xeropthalmia (dry eyes), respectively (1). It is well-established that B-cell hyperactivity is a hallmark of the disease as evidenced by altered circulating levels of B-cells, increase in serum B-cell activating factor (BAFF); hypergammaglobulinemia; and pSS-associated autoantibodies against ribonucleoproteins SS-A/Ro and SS-B/La (2). B-cell hyperactivation and local lymphocytic infiltration are also associated with the development of extraglandular manifestations which occur in a clinically relevant manner in 30–40% of pSS patients, including articular, renal, pulmonary and peripheral nervous system involvement (1).

Whilst the multi-step process leading to salivary gland (SG) loss of function remains to be established, the enhanced B-cell autoreactivity is believed to be largely driven by T-B-cell cross-talk (3). At early disease stages, antigen-presenting cells, but specifically CD4+ T-cell subsets, predominate inflammatory *foci* in the SGs (4), most likely as a result of autoantigen release by apoptotic and damaged mucosal epithelial cell activation due to viral insult, although the exact mechanism is not completely understood (5). The resulting milieu of cytokines and co-stimulation molecules in the environment activates naïve T-cells, enabling a secretion of chemokines. These act as the driving force to recruit other mononuclear lymphoid cells to the site of inflammation, including B-cells which accumulate in large numbers at later disease stages (6). In up to a third of patients, larger *foci* can develop into organized ectopic lymphoid structures (ELS), comparable to secondary lymphoid organs complete with germinal centre (GC)-like function (7).

**Figure 1:**
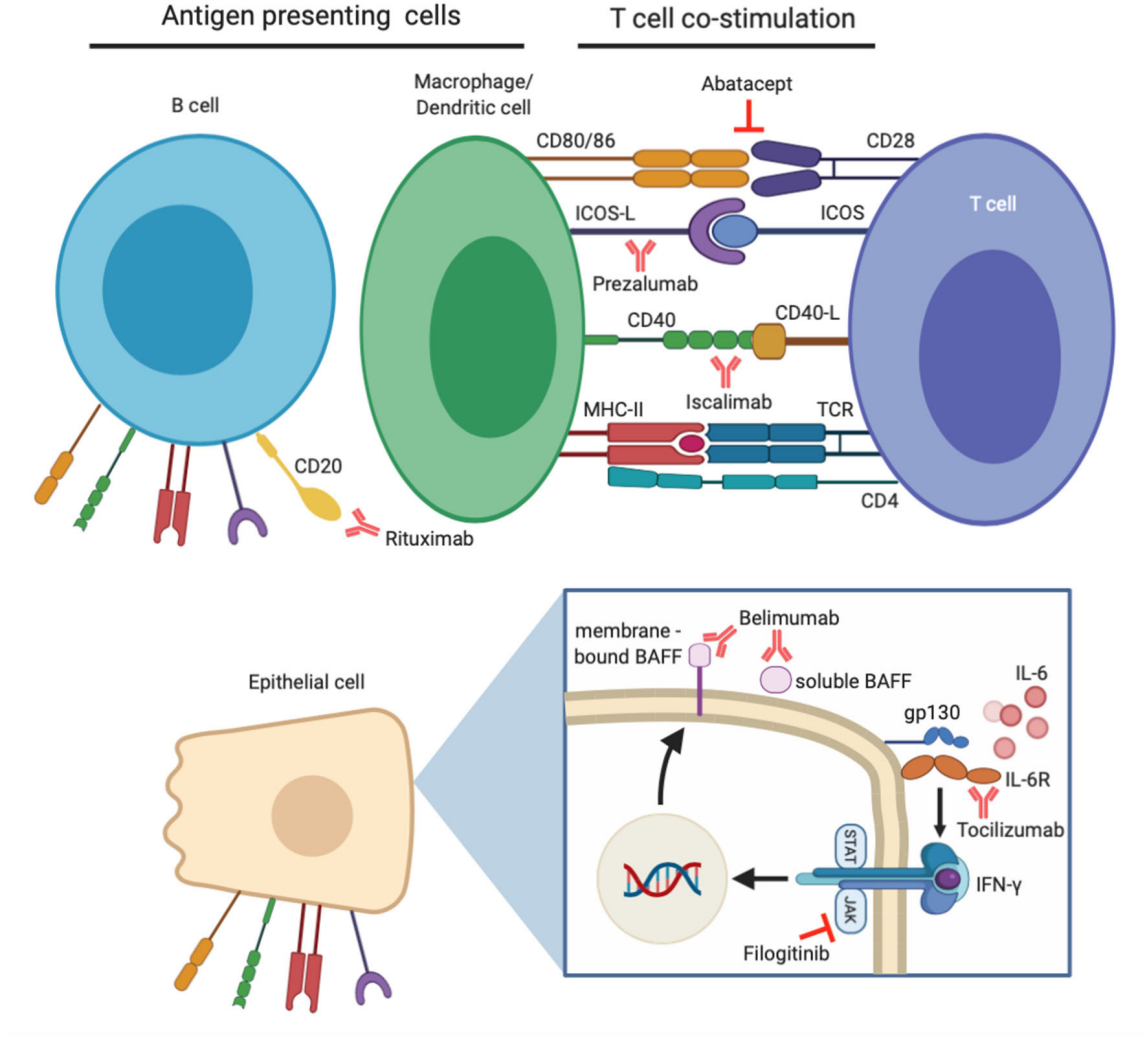
Demonstration of biological therapies clinically evaluated for the treatment of primary Sjögren's syndrome (pSS) and their target molecules. B-cell hyperactivity is a major contributor to pSS pathogenesis, and their numbers can be depleted by anti-CD20 antibody rituximab. B-cells alongside other professional (macrophages/dendritic cells) and non-professional (epithelial) antigen presenting cells are involved in enhanced T-cell co-stimulation in pSS. Specific co-stimulatory pathways can be inhibited by abatacept, prezalumab, and iscalimab. Pro-inflammatory effects of cytokine IL-6 can be prevented by tocilizumab therapy, whilst effects of IFN-γ can be mitigated by inhibition of the JAK/STAT pathway. An additional downstream effect of this is prevention of BAFF expression, which can itself be targeted by belimumab therapy.

Despite the progression in understanding pathogenic mechanisms underlying salivary gland hypofunction in pSS, current treatment options are focused on relieving symptoms rather than modifying the course of disease (8). After successful treatment in other autoimmune diseases like rheumatoid arthritis (RA), together with advances in the knowledge of pSS pathogenesis, biological compounds targeting pathways which mediate B-cell hyperactivity, T-cell co-stimulation and abnormal pro-inflammatory cytokine release are being investigated increasingly in clinical trials (Figure 1) (9). Although no biologics have yet been approved for pSS treatment, results from open label studies and randomized controlled trials (RCTs) have been promising (Table 1). In this review, we discuss key clinical and histological findings, specifically associated with salivary gland function and inflammation, reported to date from studies using biological therapy in pSS.

**Table 1:** Clinical trials which have investigated the effects of biological therapies in pSS.

**Therapy**	**References**	**Year**	**Baseline characteristics**			**Effect on SG function**		**SG inflammation**		
			**Study design**	**Cohort size (*n* =)**	**Disease duration (range) or (SD)**	**Salivary flow**	**Oral Dryness (VAS)**	**Focus score**	**Lymphocytic infiltration**	**Ultrasonography**
Rituximab	Pijpe et al. (10)	2005	Open label	15	2 ± 1	SWSF ↑ UWSF ↔	↓	N/A	N/A	N/A
	Seror et al. (11)	2007	Registry	16	9.5 (0–25)	↔	↓	N/A	N/A	N/A
	Devauchelle-Pensec et al. (12)	2007	Open label	16	13 ± 10	UWSF ↔	↓	↔	B cells ↓	N/A
	Pers et al. (13)	2007	Uncontrolled	15	N/A	N/A	N/A	N/A	B cells ↓	N/A
	Pijpe et al. (14)	2009	Uncontrolled	5	N/A	SWSF ↑	N/A	N/A	CD45+ area ↓ B: T cell ratio ↓ LEL ↓	N/A
	Meijer et al. (15)	2010	RCT	30	5 ± 4	SWSF↑ UWSF↑	↓	N/A	N/A	N/A
	Delli et al. (16)	2016				N/A	N/A	↔	B cells ↓ T cells ↔ LEL ↓ Ectopic GC ↓	N/A
	Carubbi et al. (17)	2013	Open label	41	1 (1,2)	USWF ↑	↓	↓	Chisholm and Mason grading ↓ B cells ↓ Ectopic GC ↓	N/A
	Dass et al. (18)	2008	RCT pilot	17	7 (1–18)	USWF↔	N/A		↔	N/A
	Devauchelle-Pensec et al. (19)	2014	RCT	120	5 ± 5	↔	↔	N/A	N/A	N/A
	Jousse-Joulin et al. (20)	2015				N/A	N/A	N/A	N/A	Echostructure score ↓
	Cornec et al. (21)	2016				N/A	N/A	N/A	B cells ↔	N/A
	Bowman et al. (22)	2017	RCT	133	5 ± 5	UWSF ↔	↔	N/A	N/A	N/A
	Fisher et al. (23)	2018				N/A	N/A	N/A	N/A	Total ultrasound score ↓
Abatacept	Adler et al. (24)	2013	Open label	11	6 (0.3–48)	↑	N/A	↔	Total no. of lymphocytic foci ↓ FoxP3 T cells ↓	N/A
	Haacke et al. (25)	2017	Pilot RCT	15	N/A	N/A	N/A	↔	Ectopic GC ↓ LEL ↔ Area lymphocytic infiltrate ↔ No. CD21+ FDC networks ↔ T cells ↔ B cells ↔	N/A
	Verstappen et al. (26)	2017	Open label	30	N/A	N/A	N/A	N/A	Ectopic GC ↓	N/A
	Meiners et al. (27)	2014	Pilot RCT	15	0.92 (0.58-3)	SWSF ↔ UWSF ↔	N/A	N/A	N/A	N/A
	van Nimwegan et al. (28)	2020	RCT	80	2 (1–4)	SWSF ↔ UWSF ↔	↔	N/A	N/A	N/A
	Baer et al. (29)	2020	RCT	187	5.0	SWSF ↔	↔	N/A	N/A	N/A
	Machado et al. (30)	2020	Open label	11	N/A	↑	N/A	N/A	N/A	N/A
Prezalumab	Mariette et al. (31)	2019	RCT	32	N/A	N/A	N/A	N/A	Tfh-like cells ↓ PCs ↔	N/A
Iscalimab	Fisher et al. (32)	2020	RCT	10	N/A	SWSF↑ UWSF↑	↓	N/A	N/A	N/A
Belimumab	Mariette et al. (33)	2013	Open label	30	5.7 (5.6)	UWSF ↔	↔	↔	Parotid swelling ↓	N/A
	de Vita et al. (34)	2015	Open label	30	5.9 (5.7)	UWSF ↔	↔	↔	N/A	N/A
Tocilizumab	Felten et al. (35) (abstract)	2020	RCT	110	N/A	UWSF ↔	↔	N/A	N/A	N/A

*SG, salivary gland; ESSDAI, EULAR Sjögren's Syndrome Disease Activity Index; SWSF, stimulated whole salivary flow; UWSF, unstimulated whole salivary flow; VAS, visual analogue scales; RCT, randomized controlled trial; LEL, lymphoepithelial lesions; GC, germinal centre; FDC, follicular dendritic cell; PC, plasma cells; N/A, not available*.

## Effect of B-cell Blockade Using Rituximab on Salivary Gland Function and Inflammation

The central role of B-cell hyperactivity in pSS is reflected in the American College of Rheumatology (ACR)- European League Against Rheumatism (EULAR) classification criteria, where the presence of a focal lymphocytic sialadenitis (FLS) in a labial SG biopsy (whereby a high focus score is frequently associated with large accumulation of B-cells) or positivity for circulating anti-SSA/Ro-SSB/La antibodies is mandatory in the diagnosis of pSS (36). It is of no surprise, therefore, that to date the most extensively investigated biologic in pSS, rituximab (RTX), selectively depletes B-cells. The genetically engineered chimeric IgG_1_ monoclonal antibody (mAb) is directed against the transmembrane protein CD20, which mediates B-cell activation, proliferation and differentiation (37). It is expressed on all B-cells from the pre-B-cell stage to memory B-cells and is approved for the treatment of B-cell malignancies (38–41) and other autoimmune diseases including moderate-to-severe RA (42). Initial reports of its effectiveness in pSS provided promising results, however two recent RCTs failed to meet their primary outcomes. Nevertheless, there is a general consensus that B-cell depletion in pSS is associated with a significant improvement (or lack of deterioration) of exocrine function, salivary output and reduction in inflammatory infiltrates in the SG.

The first study to test the efficacy of RTX was a single-centre phase II open label trial consisting of 15 patients. Following the standard four infusions of 375 mg/m^2^ RTX at weekly intervals, stimulated whole salivary flow (SWSF) improved significantly by the end of the trial at 12 weeks, in patients who displayed residual glandular function (>0.10 ml/min) at baseline. The study reported subjective oral and ocular dryness using the EULAR Sjögren's Syndrome Patient Reported Index (ESSPRI) which uses visual analogue scales (VAS). Oral dryness improved by the end of the trial, suggesting RTX may preserve SG function in at least a subset of patients with residual glandular activity (10). Subsequent smaller investigations reported similar improvements in PROs associated with dryness, together with reductions in parotid and submandibular SG infiltration, however this was not always reflected in objective measures of residual glandular function, measured as unstimulated salivary flow (UWSF) rate (11, 12). Importantly, RTX appeared less efficacious in trials which enrolled patients with longer disease durations, as also observed by stratifying the response in patients with early vs. long-standing disease (12).

In terms of SG inflammation, an initial study failed to report any improvement in the SG focus score (FS) (number of inflammatory *foci* of 50 cells per 4 mm^2^ area of tissue) following two cycles of therapy at 12 weeks, despite a significant depletion of B-cells in labial SG biopsies and peripheral blood (12). Sequential repopulation of B-cells was first assessed by Pers et al., and showed that after B-cell depletion was achieved in SGs at 16 weeks, this lasted for at least 12 months (13). Alongside a reduction in CD45+ lymphocyte infiltrating area, B:T-cell ratio (14) and lympho-epithelial lesion (LEL) development (16), RTX showed evidence that it may reverse FLS, or the progression of more advanced infiltrates in the form of ectopic GCs, with a reduction of their prevalence observed in multiple studies (14, 16, 17), resulting in partial restoration of SG architecture.

Based on the promising results from open label studies, one double-blind pilot study and 3 prospective RCTs have followed. The primary outcome of a 20% improvement in fatigue VAS score was not met by the pilot study, whilst secondary outcome measures of UWSF rate and changes in SG manifestations remained unchanged (18). The first positive RCT was reported by Meijer et al., where 30 pSS patients with recent and active disease were tested with RTX, meeting its primary outcome measure: a significant improvement in salivary glandular function, as measured by the SWSF rate. This was reflected in subjective PROs of sicca symptoms, highlighting clinically meaningful improvements (15). More limited benefit on SG function was reported in the TEARs RCT (Tolerance and Efficacy of Rituximab in Primary Sjogren's Syndrome, NCT00740948), which failed to improve 2 out of 4 VASs (based on global disease, pain, fatigue and dryness) by the end of the 24-week trial. Clinically significant alleviation in fatigue was achieved rapidly at week 6, and whilst marked improvements in dryness were observed at later timepoints in the treatment arm, it was less than the required 30 mm improvement using VASs (19). The largest RCT based in the UK on 133 patients, the Trial of Anti-B-cell Therapy in patients with primary Sjogren's Syndrome (TRACTISS), is the only study to introduce a second course of RTX at 24 weeks (43), when B-cell repopulation usually occurs (10, 13, 15) and to prolong therapeutic effects observed at earlier timepoints in other trials. Although TRACTISS did not achieve its primary outcome of improvements based on patient-reported VASs (30% reduction in fatigue or oral dryness) (43), a significant difference was observed between the placebo and treatment arms when assessing glandular function (22). A worsening UWSF rate in the placebo arm was observed whilst RTX maintained glandular function at weeks 36 and 48 – suggesting block of progression rather than restoration of exocrine function (43).

*Post-hoc* analyses on both TEARs and TRACTISS unveiled promising histological and morphological findings. As the first study to evaluate the effect of RTX on parotid SG morphology using ultrasonography, 50% of the TEARs cohort who underwent this procedure at a single study site significantly improved their echostructure score (decrease in the number and size of hypoechoic areas) (20). The same cohort later showed good correlation with FS (*r* = 0.61, *p* < 0.01), suggesting hypoechoic areas may present inflammatory infiltrates in the gland (44). Similar analyses on the TRACTISS cohort, this time involving multiple centres, showed significant improvement in total ultrasound score after therapy compared to placebo (23). Histologically, whilst SG inflammation measured by Chilsom and Mason grading were not originally improved in the TEARs study (19), glandular B-cell depletion in an open label cohort was observed 12 weeks after therapy, however this was not sustained in the TEARs cohort at 24 weeks (21). Combined, the effect of RTX therapy in pSS patients suggest some reversibility and restoration of SG inflammation and function.

## Effect of T-cell Co-Stimulation Blockade on Salivary Gland Function and Inflammation

Whilst pSS is often described as a disease of B-cell pathology, much of their activation is mediated by T-cell-dependent mechanisms. Antigen-presenting cells (APCs) in pSS SGs activate infiltrating naïve T-cells, to differentiate into CD4+ effector T-cells, such as T-helper 1 (Th1) and T-follicular helper (Tfh) cells to initiate an *in situ* adaptive immune response. This crucial event is mediated by activating co-stimulatory molecules expressed on APCs, namely CD80/86, CD40 and inducible T-cell co-stimulator (ICOS)-ligand, which bind CD28, CD40-ligand (CD40L) and ICOS, respectively, on T-cells. On the contrary, co-inhibitory signals can also be mediated through CD80/86 by interacting with cytotoxic T lymphocyte antigen-4 (CTLA-4) on T-cells. After antigen presentation, co-stimulation is the second signal required for T-cell activation, hence exploiting this signal could regulate perpetuation of the adaptive immune response in pSS (45).

Abatacept, a soluble fusion protein targeting CTLA-4 to inhibit CD28-mediated T-cell activation, has been approved for the treatment of RA (46) and initial studies in pSS offered promising results. Modulation of SG inflammation in the first study on abatacept was evidenced by a reduction in total number of lymphocytic foci (24). Whilst this could be due to reduced T-cell activation, the suppressive effect of Tregs can be enhanced in response to CTLA-4 blockade to enforce immunological control (47). Abatacept could impact T/B-cell co-stimulation in ectopic GCs, depleting them in parotid glands (25, 26) and attenuating Tfh-dependent B-cell hyperactivity. Abatacept stabilized glandular function in a pilot study enrolling 15 pSS patients (based on both SWSF and USWF rates), while also significantly improving systemic manifestations of disease (ESSDAI *p* < 0.001) at 24 weeks (27). Furthermore, improving ESSDAI coincided with reduced expression of T-cell activation marker ICOS, on SG Tfh cells after abatacept treatment (26). This event may be key in modulating pSS pathogenesis, since ICOS is essential for sustaining Tfh cell numbers. Disappointingly, 2 recent abatacept RCTs including the ASAP-III trial failed to meet their primary outcomes (a between-group difference in ESSDAI score at 24 weeks for both studies) with no glandular secretory improvements (28, 29). More frequent administration of abatacept coupled with longitudinal assessments could prove to be more efficacious however, as 24 monthly administrations showed systemic and functional efficacy at the end of an open label study (*p* = 0.013 median ESSDAI and salivary flow difference) (30). It is extremely important that selected primary outcomes in RCTs are sensitive to detecting change since they can be pivotal in reporting trial results, as shown by the retrospective analysis of the ASAP-III cohort, where use of a composite endpoint which considers systemic, patient-reported, functional and biological outcomes, termed CRESS (composite of relevant endpoints for SS), favored abatacept therapy over placebo (48).

In pSS, CD4+ T-cells display an activating state, including the upregulation of CD40L and ICOS costimulatory molecules to aid in B-cell activation, immunoglobulin class-switching and formation of ectopic GCs (6). Interestingly, ICOS deficiency leads to impaired CD4+ memory T-cell differentiation (49) and reduced circulating numbers of naïve, switched- and memory B-cells with pan-hypogammaglobulinemia (50), suggesting interference of this pathway may impact pSS pathogenesis. Prezalumab (MED15872/AMG557) a humanized IgG_2_ antibody targeting ICOS-L, usually expressed on B-cells and dendritic cells, was recently tested in a phase IIa study (NCT02334306). Despite proof of mechanisms evidence observed at 99 days follow-up, with SG biopsies displaying significantly fewer ICOS+ Tfh-like cells in the treatment arm compared to placebo (*p* = 0.008) and marked improvements in circulating B-cell activation markers (IgA-, IgG- and IgM-RF), the study disappointingly did not meet its primary endpoint (mean change in ESSDAI) (31). *Post-hoc* analyses on glandular function from this study is yet to be reported. To interrupt CD40-mediated T-cell-co-stimulation, Iscalimab (CFZ533) a novel antagonistic, non-depleting anti-CD40 mAb has been tested in pSS. Results from the first proof-of-concept RCT (NCT02291029) showed improvements in both stimulated and unstimulated salivary flow rates (0.04 and 0.16 ml/min, respectively) 12 weeks after treatment. Coupled with a reduction in patient-reported VAS assessments (mean decrease of 8.14 points), results from this study are promising as it is the first to show clinically meaningful improvements compared to placebo (based on ESSDAI). As of yet, ultrasonography assessment from this study are unreported, hence the assessment of Iscalimab on SG morphology is awaited (32).

## Effect of Cytokine Blockade on Salivary Gland Function and Inflammation

In pSS, a dysregulated cytokine network impairs glandular function and induces chronic inflammation systemically, making them susceptible to therapeutic targeting (51). Several cytokine families underlie the etiopathogenesis of pSS, including the interferon (IFN) family, tumor necrosis factor (TNF) family and IL-6, IL-2, IL-10, and IL-17 families (52). The type-I IFN system is induced by environmental factors in autoimmune epithelitis during early stages of disease to trigger recognition of self-antigens through pattern recognition receptors, and as such, a type-I IFN signature has been identified in pSS patients (53, 54). Binding of IFNs to their receptors activate the Janus-kinase (JAK)-signal transducer and activator of transcription (STAT) pathway, hence JAK/STAT pathway blockade could provide therapeutic benefit (52). Tofacitinib, a JAK inhibitor improved sicca symptoms in a group of patients with dry eye disease (55) whilst the JAK1 inhibitor, filgotinib has been shown to improve salivary flow in an animal model of pSS (56) and has been successful in RA treatment (57). Data describing the efficacy of JAK/STAT pathway blockade in pSS are lacking, hence results from the most recent RCT targeting this pathway with filogitinib are eagerly awaited (NCT03100942) (58).

A beneficial knock-on effect of IFN blockade through JAK1 inhibition is the downregulation of B-cell activating factor (BAFF) as observed in mice, since it is under the transcriptional control of IRF1 and IRF2 (56). BAFF [also called B lymphocyte stimulator (BLys)] belongs to the TNF family and as a critical cytokine for B-cell maturation and differentiation produced by haematopoietic and non-hematopoietic cells (59), its aberrant expression has been observed in the sera of pSS patients (60, 61) and in infiltrating SG lymphocytes (62, 63). Belimumab, a IgG_1_λ mAb neutralizing soluble BAFF has been approved for the treatment of systemic lupus erythematosus (64, 65) and is a promising treatment approach for pSS. In the proof-of-concept Belimumab in Sjogren's syndrome (BELISS) trial, a dosage of 10 mg/kg at weeks 0, 2, 4 and monthly thereafter for 6 months was evaluated and showed efficacy in 60% of the anti-SSA/B+ cohort (33). Specifically, this described a ≥30% VAS reduction in at least 2 of the following: dryness, fatigue, musculoskeletal pain, systemic activity assessed by a clinician and/or >25% improvement in any B-cell activation biomarkers. Despite significant reductions in parotid swelling and ESSDAI from baseline, improvements in SG function were not observed at 28 weeks (UWSF 0.6–0.7 mL/min, *p* = 0.27) nor were there significant changes in focus score (1.9–1.7, *p* = 0.57) (33) with similar effects observed long-term at 52 weeks (34). *Post-hoc* analysis showed belimumab restore circulating B-cell subset frequencies (66) however larger, blinded studies on belimumab are required for future trials focused on improving glandular function.

Interestingly, BAFF has been implicated in resistance to RTX. It emerged *in-vivo* that complete B-cell depletion could be sustained in mice following anti-BAFF therapy (67), suggesting a sequential, double therapeutic approach could be more effective than lone monotherapy in pSS. This biological rationale lead to the successful treatment of a pSS patient with MALT lymphoma and refractory cryoglobulinaemic vasculitis. Following failed belimumab monotherapy, the administration of RTX shortly after resulted in persistent clinical and biological efficacy, such as healing of skin ulcers, amelioration of circulating autoantibody and normalization of serum BAFF. Remarkably, a 9-year follow-up showed stable remission of lymphoma (68). Validation of the safety and efficacy of this sequential therapy has been evaluated in a multi-national pSS RCT (NCT02631538), which have now completed recruitment but the results are yet to be published.

IL-6 has a role in mediating the polarization of Tfh cells (69), B-cell activation and autoantibody production (70) and in pSS is highly expressed in serum, saliva (71) and SGs (72). Perturbing its action by tocilizumab (an anti-IL-6 receptor) has been found to be effective in RA (73) and two cases of pSS [one with refractory organizing pneumonia (74) and another with neuromyelitis optica spectrum disorder (75)]. Findings from the most recent RCT testing tocilizumab in a cohort of pSS patients (NCT01782235) has failed to meet its primary endpoint with no response observed at week 24. There was no effect on UWSF or circulating immunoglobulins or complement, suggesting that IL-6 may not be the main driver of B-cell hyperactivity in pSS (35). Limiting the primary outcome to a 3-point reduction in ESSDAI, however, lead to a high placebo effect, reinforcing the need to validate this outcome measure for use in RCTs. Other reasons for variability in the efficacy of cytokine-targeted biologics could be due to the redundant targeting of single candidates, as it may not restore all inflammation-induced damage, given the complexity of pSS pathogenesis. Exploration of alternative combination therapies could result in both clinical and biological efficacy in pSS.

## Conclusion

Based on our understanding of pSS pathogenesis and the mechanisms involving B-cells, T-cell co-stimulation and the complex network of cytokines, biologics have increased the armamentarium used for the potential treatment of this disease. Whilst this mini-review highlights promising results from recent RCTs, controversial data have made it clear that there is an urgent need to further our understanding of biological therapy in this field. For example, the failure to achieve direct and consistent clinical benefits may be overcome by exploring combinatorial therapies, taking a more holistic approach to pSS treatment. RCTs must also be well-designed and apply realistic, achievable primary outcome measures that are sensitive to change and consider the subjective, heterogenic nature of the clinical manifestations observed. Whilst ESSDAI assesses systemic disease activity, there are limitations to its use as an endpoint in RCTs (76, 77), hence progression is being made by the IMI2-NECESSITY consortium (https://www.imi.europa.eu/projects-results/project-factsheets/necessity) for the development of a reliable and validated composite outcome measure to be used in future pSS clinical trials. These changes, together with increased understanding of pathogenetic pathways could identify novel targets to be exploited by future biological therapy.

## Author Contributions

All authors listed have made a substantial, direct and intellectual contribution to the work, and approved it for publication.

## Conflict of Interest

The authors declare that the research was conducted in the absence of any commercial or financial relationships that could be construed as a potential conflict of interest.
